# Anti-quorum Sensing Activities of Selected Coral Symbiotic Bacterial Extracts From the South China Sea

**DOI:** 10.3389/fcimb.2018.00144

**Published:** 2018-05-08

**Authors:** Zhi-Ping Ma, Yu Song, Zhong-Hua Cai, Zhi-Jun Lin, Guang-Hui Lin, Yan Wang, Jin Zhou

**Affiliations:** ^1^Shenzhen Public Platform for Screening and Application of Marine Microbial Resources, The Graduate School at Shenzhen, Tsinghua University, Beijing, China; ^2^The Department of Earth Science, Tsinghua University, Beijing, China; ^3^Biology, Shenzhen Polytechnic, Shenzhen, China

**Keywords:** anti-quorum sensing, coral microbes, *S. hominis*, HPLC-MS-NMR, marine drug

## Abstract

The worldwide increase in antibiotic-resistant pathogens means that identification of alternative antibacterial drug targets and the subsequent development of new treatment strategies are urgently required. One such new target is the quorum sensing (QS) system. Coral microbial consortia harbor an enormous diversity of microbes, and are thus rich sources for isolating novel bioactive and pharmacologically valuable natural products. However, to date, the versatility of their bioactive compounds has not been broadly explored. In this study, about two hundred bacterial colonies were isolated from a coral species (*Pocillopora damicornis*) and screened for their ability to inhibit QS using the bioreporter strain *Chromobacterium violaceum* ATCC 12472. Approximately 15% (30 isolates) exhibited anti-QS activity, against the indicator strain. Among them, a typical Gram-positive bacterium, D11 (*Staphylococcus hominis*) was identified and its anti-QS activity was investigated. Confocal microscopy observations showed that the bacterial extract inhibited the biofilm formation of clinical isolates of wild-type *P. aeruginosa* PAO1 in a dose-dependent pattern. Chromatographic separation led to the isolation of a potent QS inhibitor that was identified by high-performance liquid chromatography-mass spectrometry (HPLC-MS) and nuclear magnetic resonance (NMR) spectroscopy as DL-homocysteine thiolactone. Gene expression analyses using RT-PCR showed that strain D11 led to a significant down-regulation of QS regulatory genes (*lasI, lasR, rhlI*, and *rhlR*), as well as a virulence-related gene (*lasB*). From the chemical structure, the target compound (DL-homocysteine thiolactone) is an analog of the acyl-homoserine lactones (AHLs), and we presume that DL-homocysteine thiolactone outcompetes AHL in occupying the receptor and thereby inhibiting QS. Whole-genome sequence analysis of *S. hominis* D11 revealed the presence of predicted genes involved in the biosynthesis of homocysteine thiolactone. This study indicates that coral microbes are a resource bank for developing QS inhibitors and they will facilitate the discovery of new biotechnologically relevant compounds that could be used instead of traditional antibiotics.

## Introduction

The rising problem of microbial resistance to current antibiotics and high spreading rate of resistant bacterial species has become a major public health concern. Multidrug-resistance is the biggest challenge facing the healthcare sector field (Adonizio et al., 2008). Biofilm formation is one of the mechanisms used by bacteria for developing such resistance (Vuotto et al., 2014; Arendrup and Patterson, 2017). Biofilms can act as protective membranes and are difficult to eliminate, leading to both therapy failure and disease recurrence. In recent years, it has become apparent that improved strategies and new antimicrobials are urgently needed to control infectious diseases.

Biofilm formation is controlled by cellular signals, widely known as quorum sensing (QS). Inhibition of QS is one of the many different strategies deployed to control biofilm-forming microbes without causing drug resistance (Singh et al., 2013, 2016). Some opportunistic pathogens, such as *Serratia marcescens* and *Pseudomonas aeruginosa*, control production of their virulence factors including biofilm formation by using QS systems. For example, more than 6% of the genes in the genome of *P. aeruginosa* are regulated by QS and are involved in the control of pathogenesis (Schuster et al., 2003; Wagner et al., 2003). Therefore, much work has focused on targeting microbial pathogenesis by inhibiting QS or biofilm formation. This paradigm is neither bactericidal (it does not kill bacteria) nor bacteriostatic (it does not inhibit bacterial growth). It appears to be a particularly attractive alternative to other methods because it does not impose a strong selective pressure, and thus bacterial resistance is less likely to develop (Sommer et al., 2013). For this reason, the identification of compounds that interfere with QS systems is of considerable interest in an effort to develop treatments against biofilm-associated pathogens (Christensen et al., 2007). For this reason, an approach known as QS inhibition has been developed when an efficient screening for anti-QS agents is required.

In recent years, several anti-QS compounds were reported from plants and microbes (Choo et al., 2006; Ni et al., 2009; Kalia and Purohit, 2011; Kalia, 2012). A lot of bacteria and metabolites isolated from terrestrial environments have shown anti-QS properties that can decrease the expression of virulence factors produced by some pathogens (Okuda, 2005; Adonizio et al., 2008; Tolmacheva et al., 2014). Numerous reports are emerging that provide evidence demonstrating anti-QS activity from various land sources including plants, animal extracts, fungi, and host-associated bacteria (Jiang and Li, 2013; Defoirdt, 2017; Singh et al., 2017).

Interestingly, the ocean contains a rich microbial biodiversity in which plenty of bioactive compounds are produced by various aquatic microbes, indicating that the marine environment can serve as an important resource in the search for novel anti-QS substances (Dobretsov et al., 2009; Teasdale et al., 2011; Yaniv et al., 2017). Taking coral as an example, it contains an enormous diversity of microorganisms, which render the coral microbiota ideally suited to the search for new ecological functions and bioactive metabolic compounds (Pham et al., 2016). In previous studies, the bacterial species *Oceanobacillus profundus* was isolated from the octocoral *Antillogorgia elisabethae* and was reported for its anti-QS activity by yielded compounds tyrosol and tyrosol acetate (Martínez-Matamoros et al., 2016). In addition, *Marinobacter* sp. and a Proteobacteria associated with corals have also been reported to inhibit the QS-dependent virulence factors in an environmental isolate of *S. marcescens*, which further augmented our interest in exploring coral-associated bacterial isolates (Kvennefors et al., 2012). The likelihood of finding novel bioactive compounds from coral ecosystems seems high since many such symbiotic microorganisms in this ecosystem have not been well-characterized. With this milieu, the coral ecosystem, a hitherto under-explored reserve for novel bacteria, was screened for anti-QS producers. These bacteria were then evaluated for their anti-biofilm activity, with the hope that biomolecules from such novel bacteria will be of a new and unique type.

It is worth noting that despite the abundance of active compounds from marine environments, to date the discovery and isolation of anti-QS compounds from these sources has been slow compared with the synthetic chemistry approach or terrestrial counterparts (Dobretsov et al., 2011; Yaniv et al., 2017). More importantly, a detailed identification of compounds has still not been performed (Bakkiyaraj et al., 2012, 2013). The present study stresses the importance of the coral-associated bacteria as a potential model for naturally occurring products with anti-QS properties. More specifically, given the limited knowledge available on the production of these cues by coral bacteria, the purpose of this study was to gain a clearer understanding of the ecological role of the anti-QS substances secreted by coral-symbiotic microbes.

In this study, we take the coral *Pocillopora damicornis* as the material to screen for QS-inhibiting bacteria, and one isolated bacterium was further explored for anti-QS potential. The active compounds from this bacteria were identified, expression of regulatory key genes was analyzed, and a possible mechanism of action was inferred.

## Materials and methods

### Bacterial strains and culture conditions, and coral samples

*Chromobacterium violaceum* ATCC®. 12472™ and *Pseudomonas aeruginosa* PAO1 were used in this study. Both strains were cultured in lysogeny broth (LB) medium containing 1% peptone, 0.5% yeast extract, and 0.5% NaCl, either in liquid form or solidified using 1.5% agar as necessary.

Coral (*Pocillopora damicornis*) samples were collected from Xishan Islands (located at 3°57.058′E, 36°8.532′S) in the South China Sea. The samples were collected from six sites (three from Heilong Island and three from Daming Island) at a depth of 5–6 m. The salinity was around 33.1‰ (33.1 per thousand) and the seawater temperature was 29.7°C. At each site, five coral samples were collected. Samples were washed with sterile seawater, homogenized by grinding and agitation, and serially diluted in sterile seawater. Next, 50 μl of dilutions from 10^−4^ to 10^−7^ were surface-plated on marine agar 2216 (Difco, USA) and incubated at 30°C for 3–5 days. A quantity of pre-test colonies, chosen on the basis of their different colonial morphology, were collected by sterile toothpick and incubated in the conditions described above.

### Screening and identifying anti-QS bacteria

A disc diffusion assay (Bauer et al., 1966) was performed with biosensor strain *C. violaceum* ATCC 12472 to detect anti-QS activity (Busetti et al., 2014). Briefly, 5 ml overnight reporter strain culture is poured into 45 ml LB media containing 0.75% agar until the temperature of the media is about 45°C. The mixture is then plated and allowed to solidify before sterile filter paper circles (5 mm diameter) are placed on the LB surface at regular intervals. The screened single colony isolates are cultured overnight in LB medium at 30°C in 1.5 ml Eppendorf tubes with constant shaking at 150 rpm. The cultured individual as the test strains (OD_600_ near 0.1) and bacterial suspension (3 μl) are pipetted onto the filter paper. 2,5-Dimethyl-4-hydroxy-3[2H]-furanone (CAS No. 3658-77-3, Sigma-Aldrich, USA) dissolved in dimethyl sulfoxide (DMSO, 1 μl), DMSO solvent, and LB broth are used as positive, negative and blank controls, respectively, in this plate-based bioassay. After incubation for 24 h at 30°C, inhibition of pigment production around the disc (a colorless ring) is checked. Positive anti-QS activity will be recorded as visible colorless haloes like furanone. The bacterial isolates showing promising positive anti-QS activities are selected for further study. To ensure reliability of the experiment, the anti-QS activities of the selected isolates are repeated three times independently.

Potential anti-QS strains were grown overnight in LB broth at 30°C, and then 200 μl from each culture was transferred into a clean 1.5 ml Eppendorf tube and centrifuged at 7,000 g for 1 min (Chang et al., 2017). The flow-through in the tube was discarded, 100 μl TE buffer was added, and the sample was mixed gently, and then boiled for 10 min. The resulting supernatant contained the crude DNA extract (OD_260_/OD_230_ was more than 1.7, and OD_260_/OD_280_ was between 1.8 and 2.0). The 16S rRNA gene, which is approximately 1500 bp, was amplified by PCR using the forward primer 27F (5′-AGAGTTTGATCCTGGCTCAG-3′) and the reverse primer 1492R (5′-GGTTACCTTGTTACGACTT-3′) (Lane, 1991), and sequenced at BGI-Shenzhen (BGI China, Mainland). The sequences obtained were assembled, analyzed, and manually edited using the CAP3 software package. The resulting sequences were compared against those from the NCBI database (http://www.ncbi.nlm.nih.gov) using BLAST analysis and the RDP online service (https://rdp.cme.msu.edu).

### Determination of growth and violacein production

The effect of the potential anti-QS bacterial extract on the growth of *C. violaceum* ATCC 12472 was determined by the colony count on plate method (Choo et al., 2006). Cultures of *C. violaceum* ATCC 12472 were serially diluted and 100 μl aliquots were spread on LB plates. The plates were incubated at 30°C for 24 h, and bacterial counts were compared with the control. For quantification of violacein production, 1 ml of culture was centrifuged at 13,000 rpm for 10 min to precipitate insoluble violacein. The culture supernatant was discarded and 1 ml DMSO was added to the pellet. The solution was vortexed vigorously for 30 s to completely solubilize violacein and was then centrifuged at 13,000 rpm for 10 min to remove cells (Choo et al., 2006). Two-hundred microliters of the violacein-containing supernatants were added to 96-well flat-bottomed microplates, three wells per sample, and the absorbance was read with a spectrophotometer (Infinite® 200 PRO, Tecan, Austria) at a wavelength of 585 nm (Blosser and Gray, 2000).

### Extracting the anti-QS active components

The possible anti-QS strains were incubated for 48 h in LB broth at 30°C with shaking at 200 rpm. Samples were then centrifuged at 6,000 g, 4°C for 20 min to remove bacterial cells, and the resulting supernatants were collected and extracted using an equal volume of ethyl acetate, with vigorous shaking for 15–20 min. The extraction was repeated twice and the aqueous extract fractions were discarded. The organic extract fractions (obtained by ethyl acetate extraction) were combined and evaporated in a rotary evaporator at 45°C. The organic residues were dissolved in methanol (Nithya et al., 2010c) and concentrated using nitrogen flow. All resulting extracts were sterilized using 0.22 μm filters.

A second-round of testing for anti-QS activity was carried out using the sterilized extracts and following the above-mentioned methods (see “Screening and identifying anti-QS bacteria”). The putative extracts (from the anti-QS strains identified in the preliminary screen) were pipetted onto the filter paper, and the QS inhibition activity was calculated by measuring the diameter of colorless haloes relative to equivalent furanone. Finally, the positive extracts were stored at −20°C and used for biofilm inhibition experiments at a range of concentrations.

### Influence of anti-QS extract on *P. aeruginosa* PAO1 biomass and cellular growth

The effects of the extract from the anti-QS positive strain on the biomass of biofilms produced by *P. aeruginosa* PAO1 were determined using the crystal violet (CV) method (Huber et al., 2003; Choo et al., 2006). Briefly, freshly cultured *P. aeruginosa* PAO1 was added to 96-well polystyrene plates (100 μl per well) and incubated in LB medium (Hinsa, 2006). The bacterial extracts (for example D11 strain) were added at 1, 2.5, 5, and 10 μg/ml (w/v). The mixtures were incubated at 30°C for 48 h. Planktonic cells and spent medium were removed from each culture. The remaining adherent cells were gently rinsed twice using deionized water. One-hundred microliters of 1% (w/v) CV solution was added to each well for 30 min at room temperature. The excess dye was discarded, and the plates were washed gently but thoroughly using deionized water. The CV-stained cells were solubilized in DMSO and the absorbance at 600 nm was determined using a microplate reader (Infinite® 200 PRO, Tecan). *P. aeruginosa* PAO1 cultures incubated in the absence of extract and lose QSI ability extract (ultrasonic method to destroy the chemical structure of the extract) served as negative controls. Pure water was used as a blank control. Experiments were performed with 12 replicates (12 replicate wells in 96-well plates) for each treatment. When absorbance was determined, three readings were recorded for each well.

To determine the effects of the extract on the growth of *P. aeruginosa* PAO1, a growth curve assay was conducted. *P. aeruginosa* PAO1 was cultured in LB broth in the presence or absence of extract from strain D11 (10 μg/ml, w/v). Cultures were incubated at 30°C for 48 h. After 0, 3, 6, 9, 12, 18, 27, 36, and 48 h, the optical density at 600 nm was determined using a microplate spectrophotometer (Infinite® 200 PRO, Tecan). Bacterial abundance was measured using a flow cytometer (BD Biosciences, USA). Briefly, samples (1 ml each) were fixed with glutaraldehyde (0.5% final concentration), then stained with SYBR green I solution (Molecular Probes) (at a 1000-fold dilution of the stock solution) at room temperature in the dark for 15 min (Gasol and del Giorgio, 2000). Fluorescent 1-μm latex beads (10^5^ beads per ml) were added to the samples as an internal standard. Bacterial number (cells/ml) were calculated by their signatures in a side-scatter-vs.-green-fluorescence plot, as described by Pinder et al. (1990) and Gasol and del Giorgio (2000).

### Separation and identification of anti-QS active compounds

Extract compounds were separated by preparative high-performance liquid chromatography (HPLC) (Agilent 1200, USA). Samples were kept at 4°C until injection, and 100 μl extract sample was injected onto a reverse-phase C18 core-shell column (50 × 2.1 mm, Waters, CA, USA) via an auto-sampler (ThermoFisher Scientific, USA). The mobile phase was obtained using 83% methanol and 17% water at a flow rate of 0.5 ml/min at 30°C. Samples, separated every 30 s, were collected with the fraction collector. At the end of the separation process, every peak sample was taken and concentrated by nitrogen-flow method. Every purified sample was then re-tested to confirm the anti-QS activity using the procedures described above (see “Screening and identifying anti-QS bacteria”).

The collected anti-QS active peaks were further purified on HPLC (Waters Delta Prep 4000, USA) using a C18 column and a linear water/acetonitrile gradient containing 0.1% trifluoroacetic acid. The residue was dissolved in 1 ml acetonitrile/water (1:1, v/v) to determine the molecular weight by mass spectrometry (MS) on a LTQ XL Orbitrap using a static nanospray (Thermo-Fisher, CA, USA) in positive-/negative-ion mode. To determine the active molecular structure, nuclear magnetic resonance spectroscopy (NMR) was performed on the purified anti-QS active sample to get a heteronuclear single quantum coherence (HSQC) and heteronuclear multiple bond coherence (HMBC) spectrogram.

### Inhibition of biofilm

Pre-sterilized glass microscope slides were used to observe biofilms by confocal laser scanning microscopy (CLSM) as described in the previous study (Ortlepp et al., 2007; Tolker-Nielsen and Sternberg, 2014). Briefly, *P. aeruginosa* PAO1 was grown in LB medium overnight and diluted with fresh medium to an OD_600_ of about 0.02. Then, 2 ml dilutions were incubated under static conditions with or without anti-QS extract (10 μg/mL, w/v) in 12-well plates with a glass microscope slide in each well. After 12 and 36 h, the glass slides were gently lifted out and rinsed with deionized water to remove loosely attached cells. The biofilms on one side were stained with 5 μM SYTO9 dye (Sigma, USA) in the dark, and those on the other side were wiped off. After 15 min, the slides were washed, and observed by CLSM (Zeiss, Germany) with a × 60 objective lens to visualize the biofilms. The 488 nm excitation and 520 nm emission filter settings were used for detection of SYTO9. Quantification of biofilm parameters was processed with the COMSTAT software using the CLSM images (Heydorn et al., 2000). Of the available parameters, we selected the three factors of total biomass, average thickness, and roughness coefficient to evaluate the biofilms (Hentzer et al., 2001). 3D transmission-fluorescence photos of the *P. aeruginosa* PAO1 biofilms were produced using FV10-ASW2.0 Viewer (Olympus, Japan). The optical sections were 5 μm apart on the Z-axis and taken at 640 × 640 pixels with a 12-bit intensity resolution (Chang et al., 2017). Digital images were processed using Leica Confocal Software Lite (Leica Microsystems, Germany).

### Effect of anti-QS extract on the expression of QS genes

*P. aeruginosa* PAO1 was grown in 10 ml LB liquid medium to an OD_600_ of approximately 0.1. At this time point, treated groups had approximately 10 μl extract added (extract concentration was 10 μg/ml) to the *P. aeruginosa* PAO1 culture medium. The extract was dissolved in methanol and the final methanol concentration in the experimental system was 0.1% (v/v). Solvent control groups had 10 μl methanol only added. After 24–36 h, total RNA was extracted from control and treated groups using RNAiso Plus Reagent (Takara, China), and reverse-transcribed into cDNA with PrimeScript RT reagent kit (Takara) according to the manufacturer's protocol. Before performing the quantitative real-time PCR (qRT-PCR), RNA quality was determined (by measuring A_260_/A_280_ and A_260_/A_230_, and by gel electrophoresis). Eight reported functional genes coding for QS regulation activity were chosen for PCR analyses. Primers were designed using Primer Express 3.0 (Applied Biosystems) and are listed in Table 1. Thirty-two PCR cycles were run with denaturation at 95°C for 15 s, annealing at 55°C for 30 s, and extension at 60°C for 45 s. The 16S rRNA gene was used as a control for standardization. A melt curve analysis was also done for the validation of specificity of the qRT-PCR. The relative transcription level of each gene was defined as the ratio of its transcript of biofilms grown in the indicated concentration of compounds over that in LB medium with methanol, using the 2^−ΔΔCt^ method (Livak and Schmittgen, 2001).

**Table 1:** Primers for quantitative reverse transcriptase-PCR.

**Gene**	**Forward primer (5′−3′)**	**Reverse primer (5′−3′)**
*rhlR*	AACGCCAGATCCTGCAATG	CGGCGTCGAACTTCTTCTG
*rhlI*	GCAGCTGGCGATGAAGATATTC	CGAACGAAATAGCGCTCCAT
*lasR*	GACCAGTTGGGAGATATCGGTTA	TCCGCCGAATATTTCCCATA
*lasI*	GCCCCTACATGCTGAAGAACA	CGAGCAAGGCGCTTCCT
*lasA*	GACCAGTTGGGAGATATTAGTTA	TCCAAAGAATATTTCCCATA
*lasB*	CGACAACGCGTCGCAGTA	AGGTAGAACGCACGGTTGTACA
*pqsA*	AACGCCAGATCCTGCAATG	CGGCGTCGAACTTCTTCTG
*pqsR*	GCAGCTGGCGATGAAGATATTC	CGAACGAAATAGCGCTCCAT

### Whole-genome sequencing of strain D11

Genomic DNA of strain D11 was extracted using a GenEluteTMkit (Sigma-Aldrich, USA) and converted into a next-generation sequencing library using Next-era XT (Illumina, CA, USA) according to the manufacturer's instructions. Whole-genome sequencing was performed using the MiSeq at BGI Company (Shenzhen, China). SMRT Analysis 2.3.0 was used to filter low-quality reads and the sequences were assembled using Spades v2.5 (default setting) (Bankevich et al., 2012). The generated contigs were scaffolded and gap-closed using SSPACE and GAPFiller, respectively (Boetzer et al., 2011; Boetzer and Pirovano, 2012). Genome annotation was performed using Prokka and InterProScan5 (Jones et al., 2014; Seemann, 2014).

The software tRNAscan-SE v.1.23 and RNAmmer v.1.2 were used to identify presence of tRNA and rRNA, respectively (Lagesen et al., 2007). Gene prediction was performed by GeneMarkS with an integrated model that combined the GeneMarkS generated (native) and heuristic model parameters (Besemer et al., 2001). A whole-genome BLAST search (*E*-value less than 1 × 10^−5^), minimal alignment length percentage larger than 40%, was performed against the main databases, including KEGG (Kyoto Encyclopedia of genes and genomes), COG (Clusters of Orthologous Groups), and Swiss-Prot. The annotation predictions were manually evaluated and only genes predicted with consensus from two or more annotation pipelines were trusted in order to provide gene identification with high confidence.

### Statistical analysis

Differences in various data were determined using analysis of variance (ANOVA) at the *P* < 0.05 significance level. All analyses were performed using the SPSS software package 13.0 (NY, USA).

## Results

### Isolation and identification of anti-QS coral bacteria

**Figure 1:**
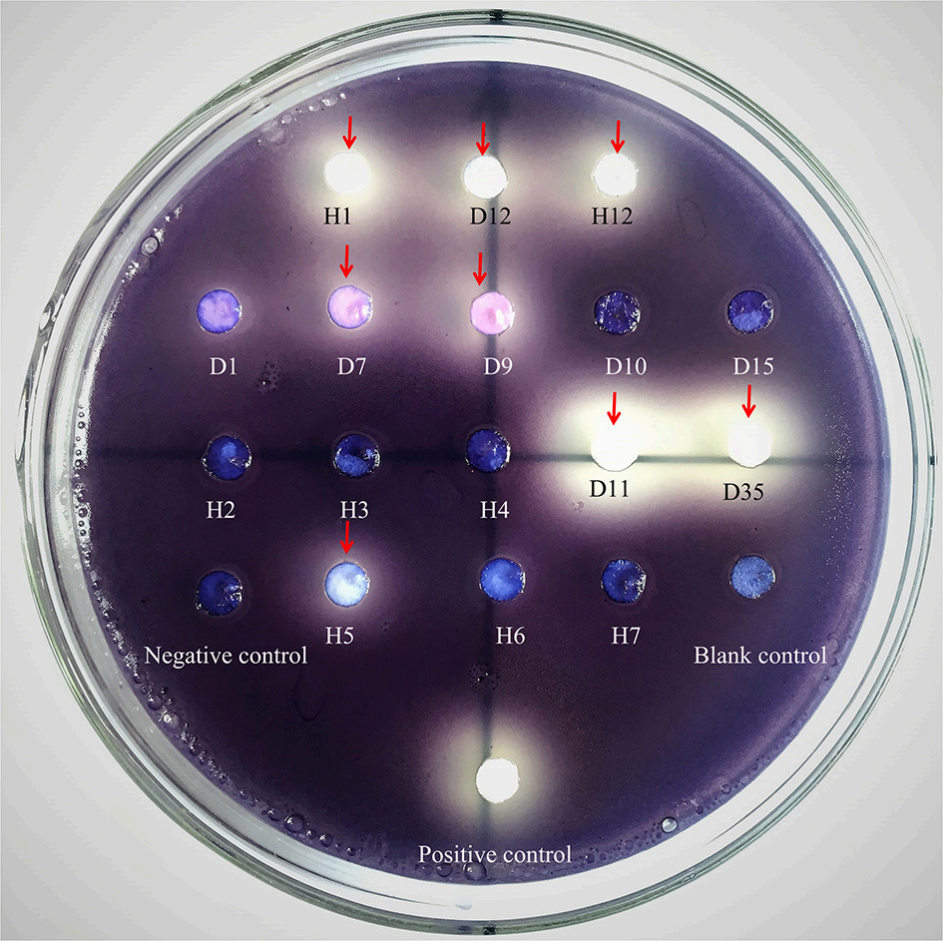
Screening of anti-QS strains on biosensor plates containing reference strain *C. violaceum* ATCC 12472 and filter paper for sample detection. Water, LB medium and furanone (diluted 10 times with DMSO) were used as blank, negative and positive controls, respectively. The absence of purple or formation of a pigment inhibition was considered to indicate a potential QS inhibitor. The red arrows refer to the positive anti-QS strains and the pigment inhibition can be observed on a clear background on the plate. Notes: the number indicated the test isolate strains. H samples come from Heilong Island, and D samples come from Daming Island.

The possible anti-QS bacteria were screened using *C. violaceum* ATCC 12472 as an indicator strain since it produces the purple pigment violacein unless its QS system is interrupted. Using this technique, a lack of pigmentation from the indicator organism in the vicinity of the test organism indicates a potential anti-QS result (do Valle Gomes and Nitschke, 2012). A total of 200 culturable bacteria were isolated from the *Pocillopora damicornis* symbiotic environment and screened for anti-QS ability. About 15% (30 isolates) were positive in the screen for color reduction in *C. violaceum* ATCC 12472, with representative results shown in Figure 1. Some isolates showed promising anti-QS activity and a distinct white opaque zone of inhibition was observed in the biosensor plate containing reference strain *C. violaceum* ATCC 12472. The activity of positive isolates was recorded as either strong, medium or weak, based on the diameter of visible colorless haloes by the biosensor (Table 2). The isolate D11 caused the most significant reduction (the diameter of visible colorless haloes is 18.36 mm), in which the purple pigment of *C. violaceum* ATCC 12472 was completely eliminated (Figure 1). In comparison, the zone of inhibition was not detected with the negative control (DMSO solvent) or blank control (LB medium only).

**Table 2:** Anti-QS activity of selected coral symbiotic bacteria and taxonomical identification.

**Isolate no**.	**Bacterial species**	**% Identity**	**Anti-QS activity (diameter of the white opaque zone, mm)**
D11	*Staphylococcus hominis*	100	18.36
D35	*Staphylococcus warneri*	99	13.08
H1	*Lysinibacillus fusiform*	99	10.39
D12	*Bacillus cereus*	99	9.84
H12	*Vibrio alginolyticus*	98	11.25

*Differences in diameter of the white opaque zone indicate differences in anti-QS activity. Notes: in this study, 30 potential anti-QS strains were screened. After16S rRNA gene sequencing, five poor quality sequences were removed. The remaining25 high quality sequences were subjected to BLAST searches in the NCBI database; after dereplication, five bacteria were successful identified and are shown in this table*.

The 16S rRNA gene sequences of these 30 positive isolates were aligned to the NCBI database using BLAST. Most of the representative isolates shared 99% sequence similarity with their respective reference strains. After filtering low-quality sequences and dereplication analyses, five representative strains were chosen from the candidates for anti-QS active substance studies. These five bacterial strains were *Staphylococcus hominis, Lysinibacillus fusiform, Bacillus cereus, Staphylococcus warneri*, and *Vibrio alginolyticus*. The 16S rRNA gene sequences for these five strains have been submitted to the GenBank database under the accession numbers MG761744–MG761748. Among the five strains, isolate D11 revealed a 100% sequence similarity to *Staphylococcus hominis* and has been tentatively named *S. hominis* D11 (GenBank accession number is MG761745). In the following experiment, we chose *S. hominis* D11, which has the most anti-QS activity, as the research object.

### Effect of anti-QS extract on growth and violacein production of *C. violaceum* ATCC 12472

**Figure 2:**
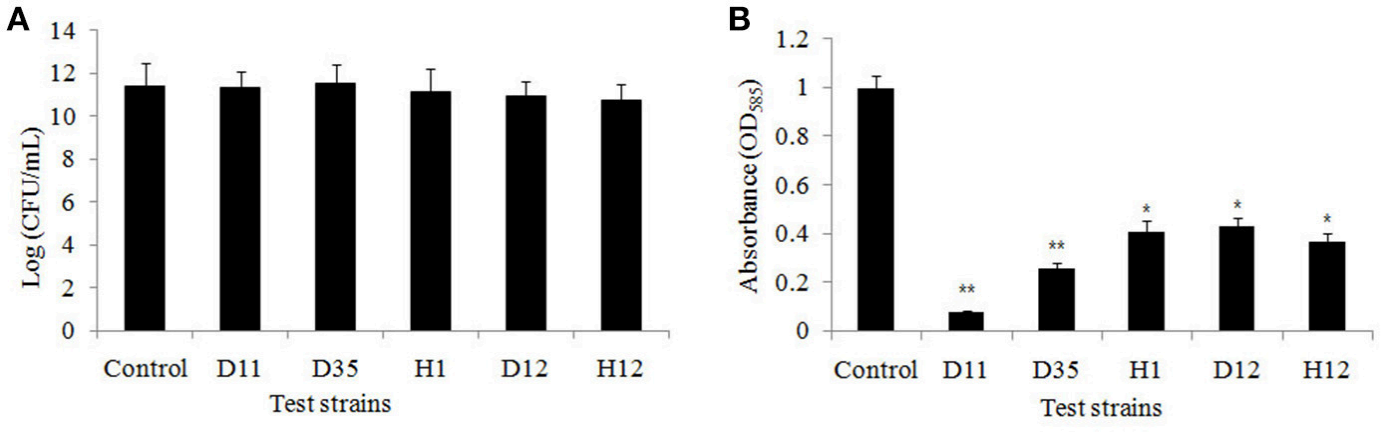
**(A)** Bacterial cell count of the flask incubation assay. The five test isolates were D11 (*Staphylococcus hominis*), D35 (*Staphylococcus warneri*), H1 (*Lysinibacillus fusiform*), D12 (*Bacillus cereus*), and H12 (*Vibrio alginolyticus*). *C. violaceum* ATCC 12472 was incubated for 16 h, and 100 μl of the bacteria, adjusted to OD_600nm_ of 0.1 (approximately 1 × 10^8^ CFU/ml), were spread on LB plates. The growth inhibition were compared with control. Data are presented as the logarithm of mean CFU ± SD. **(B)** Inhibition of violacein production by test strains. Violacein production was measured spectrophotometrically as described in the Materials and Methods. Data are presented as mean ± SD of absorbance at 585 nm. Asterisks indicate a statistically difference between experimental groups and control groups (^*^P < 0.05; ^**^P < 0.01).

The results of the colony count performed on LB plates at 24 h incubation showed no significant difference in the number of colony forming units (CFU) (Figure 2A). This indicates that the tested strains (D11, *S. hominis*; D35, *L. fusiform*; H1, *B. cereus;* D12, *S. warneri*; and H12, *V. alginolyticus*) have no effect on the growth of *C. violaceum* ATCC 12472. The five bacterial isolates showed a significant drop in violacein content, especially isolate D11 where violacein production was reduced by 92.3% (Figure 2B). Therefore, reduced production of violacein by bacterial culture was not due to the reduction of the “quorum,” but due to the interruption of the “sensing.”

### Extract from strain D11 inhibits biofilm formation

**Figure 3:**
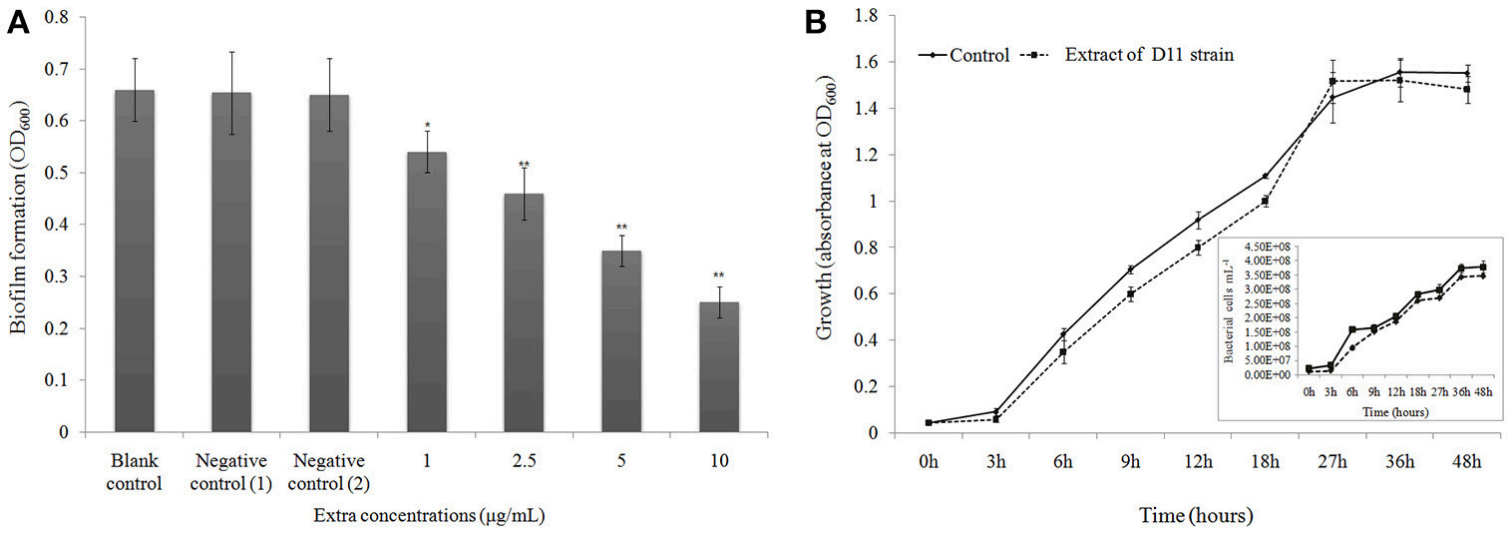
**(A)** Biofilm dispersal activity (crystal violet assay) of extract from isolate *Staphylococcus hominis* D11. Different concentrations of bacterial extract (1–10 μg/ml) were tested against the widely used biofilm-forming reference strain *P. aeruginosa* PAO1. Experiments with “extract + DMSO,” “lose QSI ability extract + DMSO,” “DMSO only,” and “no extract + no DMSO” were considered as test control, negative control (1), negative control (2), and blank control, respectively. Asterisks indicate a statistically significant difference (^*^P < 0.05; ^**^P < 0.01) between experimental groups and control groups. Data are presented as mean ± SD (*n* = 3). **(B)** Effect of anti-QS compounds on growth of *P. aeruginosa* PAO1. Bacteria were grown in LB media with (dotted line) and without (solid line) D11 strain extract (10 μg/ml). The extract did not affect specific bacterial growth rate or bacterial abundance. Flow cytometry results (inset picture) show the count of bacterial cells. Data are presented as mean ± SD (*n* = 3).

The anti-biofilm activity of the D11 extract was tested against the widely used biofilm-forming clinical isolate *P. aeruginosa* PAO1. Figure 3A presents quantitative analysis of *P. aeruginosa* PAO1 biofilm inhibition. Addition of *S. hominis* D11 extract (1, 2.5, 5, and 10 μg/ml) to *P. aeruginosa* PAO1 reduced biofilm formation by 18.2, 30.3, 46.7, and 62.1%, respectively, indicating that the inhibition occurred in a dose-dependent manner. The possibility of an inhibitory effect of the D11 extract on the growth of *P. aeruginosa* PAO1 was also analyzed. However, no significant effect on growth of *P. aeruginosa* PAO1 was observed in the presence of 10 μg/ml bacterial extracts (Figure 3B).

**Figure 4:**
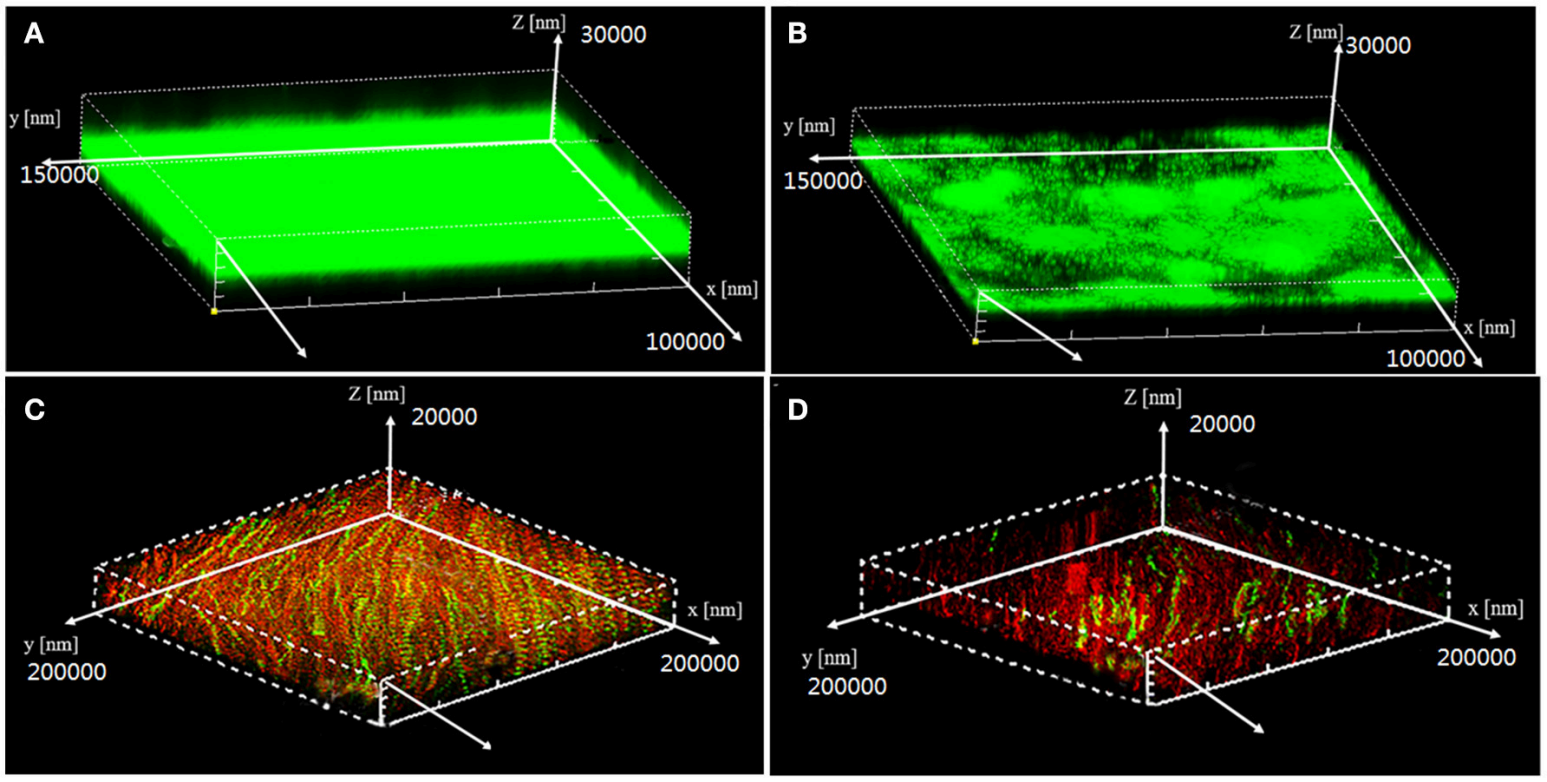
Confocal scanning laser microscopy (CLSM) z-stack 3-D images of *P. aeruginosa* PAO1 biofilm architecture in the presence (10 μg/ml)or absence (0 μg/ml) of D11 extract in media with 2% glucose. Data shown are early stage (12 h) biofilm structure of *P. aeruginosa* PAO1 in control group **(A)** and treatment group **(B)**, and the post-stage (36 h) biofilm structure of *P. aeruginosa* PAO1 in control group **(C)** and experimental group **(D)**. In these images, live bacterial cells produced green fluorescence, whereas dead cell sproduced red fluorescence.

**Figure 5:**
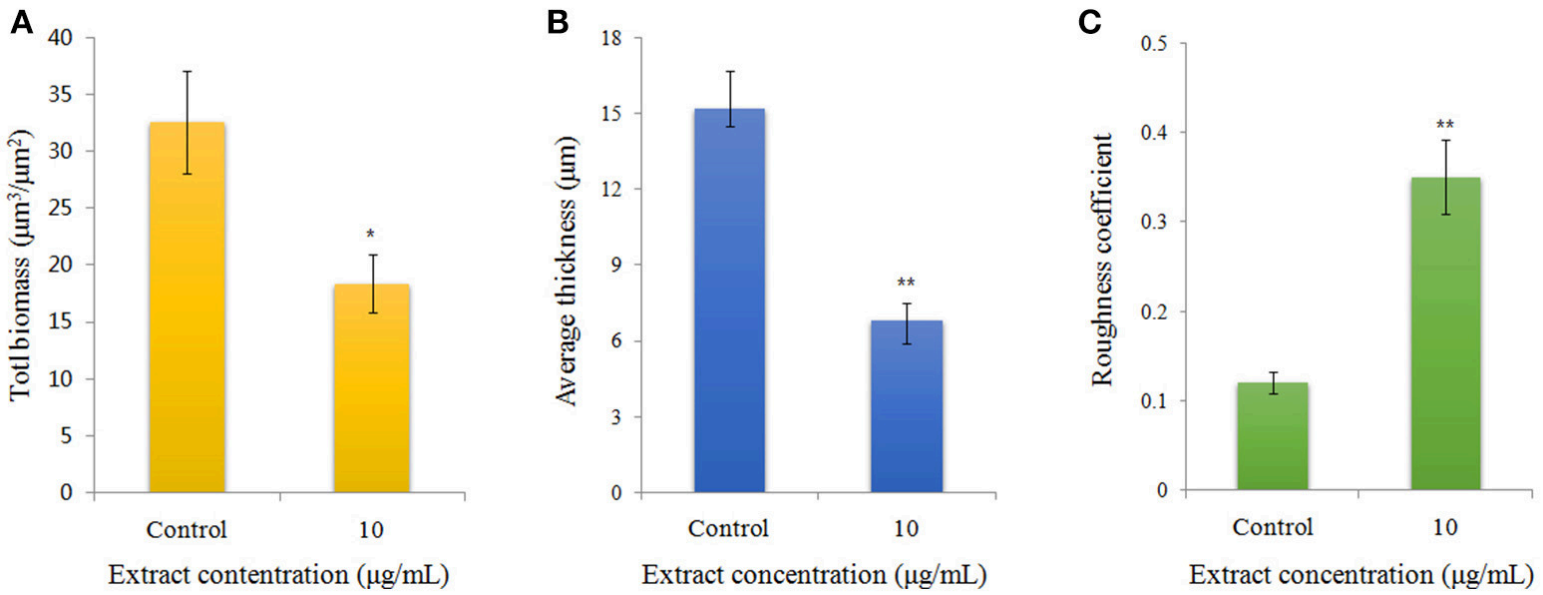
Quantification of biofilm formation of *P. aeruginosa* PAO1 (taking 36 h as example) using COMSTAT software, including **(A)** bio-volume, **(B)** average thickness, and **(C)** roughness coefficient. Error bars indicate SD (*n* = 3). Asterisks indicate a statistically significant difference (^*^P < 0.05; ^**^P < 0.01) between experimental groups and control groups.

Visualization of biofilms by microscopy analysis enabled precise evaluation of the biofilm 3D-structure. The topology of the biofilm developed by *P. aeruginosa* PAO1 and the effect of the D11 extract on it was analyzed by CLSM. A well-grown biofilm along with adhering bacterial cells was observed in control samples (normal biofilm developed by *P. aeruginosa* PAO1) at 12 and 36 h (Figures 4A,C), whereas dispersed bacterial cells were observed in treated samples (Figures 4B,D). Extremely thick biofilms (more cells and polysaccharides) were formed in the control relative to the experimental group. Also, the COMSTAT analysis clearly showed the disrupted surface topology and height distribution profile of the biofilm developed in the presence of the D11 extract compared to the control biofilm (taking36 h as the example) (Figure 5). In control groups, *P. aeruginosa* PAO1 developed a thick, dense biofilm, whereas on a surface coated with the D11 active crude extract, biofilm formation and bacterial adherence were prevented. Quantitative analysis showed that the D11 crude extract surface coating inhibited biofilm total biomass and average thickness by 43.9 and 58.7%, respectively (Figures 5A,B).

### Identification of anti-QS compounds

**Figure 6:**
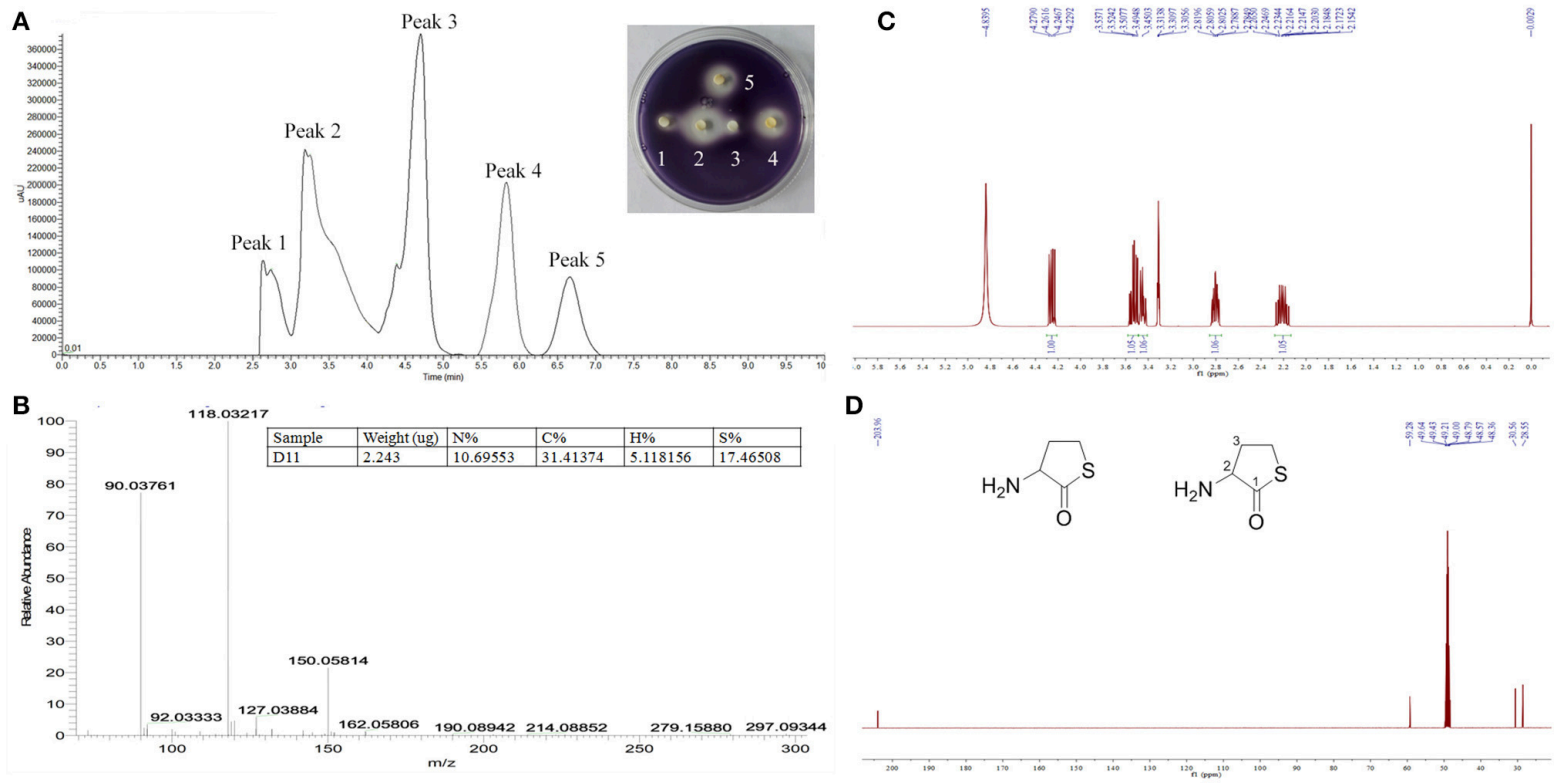
Analysis of active fraction showing anti-QS activity. **(A)** Pre-HPLC analysis of *S. hominis* D11 extract. The chromatogram shows the five main active peaks from *S. hominis* D11. The insert picture in **(A)** is the re-test of the anti-QS activity of the five peak compounds. **(B)** GC chromatograms and ESI-MS/MS of the active fraction (peak 2) of *S. hominis* D11 extract. Peaks are a function of intensity measured in milli-absorption units over time in minutes. **(C)** ^1^H-NMR spectrum of compound in Methanol-*d*_4_ at 400 MHz. **(D)** ^13^C-NMR spectrum of compound in Methanol-*d*_4_ at 100 MHz. ^1^H-NMR (Methanol-*d*_4_, 400 MHz) δ:4.25 (1H, dd, *J* = 12.9, 7.0 Hz, H-2), 3.53 (1H, td, *J* = 11.6, 5.2 Hz, H-4), 3.45 (1H, ddd, *J* = 11.6, 7.2, 1.1 Hz, H-4), 2.80 (1H, dddd, *J* = 12.2, 6.7, 5.2, 1.4 Hz, H-3), 2.21 (1H, m, H-3). ^13^C-NMR (Methanol-*d*_4_, 100 MHz) δ:204.0 (C-1), 59.3 (C-2), 30.6 (C-3), 28.6 (C-4). The insert picture in **(D)** is the structure of DL-homocysteine thiolactone (redrawn by ChemBioDraw Ultra 12.0).

Pre-HPLC analysis was applied to separate the crude extracts, with fractions collected every 30 s. The chromatogram from the liquid chromatography mass spectrometer (ThermoFisher Scientific™ TSQ Altis™, USA) showed that five main peaks exist (Figure 6A). The five fractions were collected and anti-QS activity was individually retested for each fraction using the biosensor plate containing *C. violaceum* ATCC 12472. Fraction peak 2 showed a maximum zone of QS inhibition; therefore, this fraction was selected for further characterization. Fraction peak 2 was subjected to HPLC and gas chromatography-mass spectrometry (GC-MS) analysis, and a main mass spectral peak, detected at m/z 118.03, was considered the corresponding experimental mass of the active fraction (Figure 6B). The detected mass spectra showed some resemblance to homocysteine thiolactone in the GC-MS library. The calculated (theoretical) or expected molecular mass of compound homocysteine thiolactone is 118. The molecular mass of the active fraction was further confirmed by NMR (C^13^ and H^1^) (Figures 6C,D).

**Figure 7:**
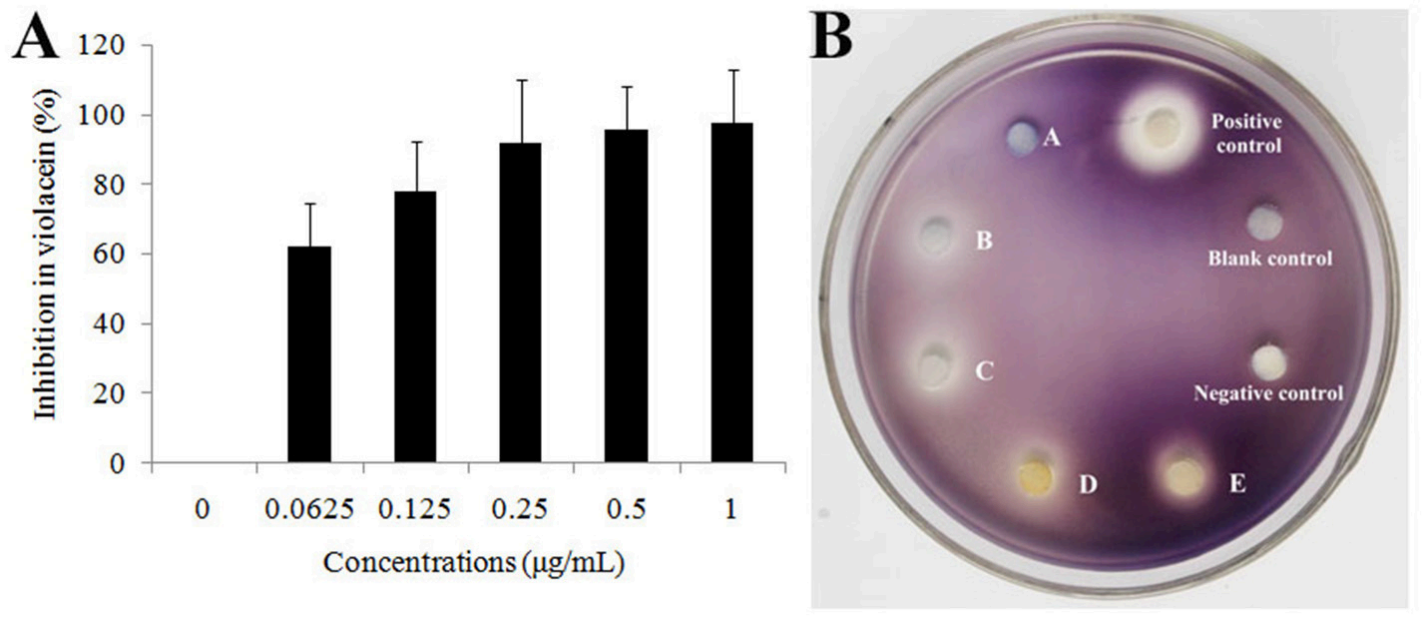
Effect of DL-homocysteine thiolactone on violacein production by *C. violaceum* ATCC 12472. **(A)** Percentage of inhibition of the violacein pigment after incubation with different concentrations of DL-homocysteine thiolactone. A dose-response effect in the production of violacein was observed in a series of concentrations (0.0625, 0.125, 0.25, 0.5, and 1.0 μg/mL) of DL-homocysteine thiolactone. Data show the mean (±SD) of three independent experiments. **(B)** Anti-QS activity of pure DL-homocysteine thiolactone at different concentrations. A, 0.0625 μg/ml; B, 0.125 μg/ml; C, 0.25 μg/ml; D, 0.5 μg/ml; and E, 1.0 μg/ml. LB medium, DMSO only and furanone (dissolved in DMSO, 1.0 μg/ml) were used as blank, negative and positive controls, respectively.

In order to confirm the QS inhibitory activity produced by strain D11 can be attributed to homocysteine thiolactone, the commercial product (DL-homocysteine thiolactone, CAS No. 6038-19-3) was purchased from the Macklin Biochemical Co., Ltd (Shanghai, China). The anti-QS activity of this commercial product was tested according to the above-mentioned methods. The inhibitory activity of DL-homocysteine thiolactone against bacterial QS was determined using violacein production by *C. violaceum* ATCC 12472. From Figure 7A, a concentration-dependent inhibitory activity was observed, with the tested concentrations (0.0625, 0.125, 0.25, 0.5, and 1.0 μg/ml) of DL-homocysteine thiolactone showing a significant inhibition in violacein content (ranged from 62.5 to 98.1%). A varying degree of white opaque zone of inhibition was also observed in the biosensor plate containing reference strain *C. violaceum* ATCC 12472 (Figure 7B).

### Expression analysis by qRT-PCR

**Figure 8:**
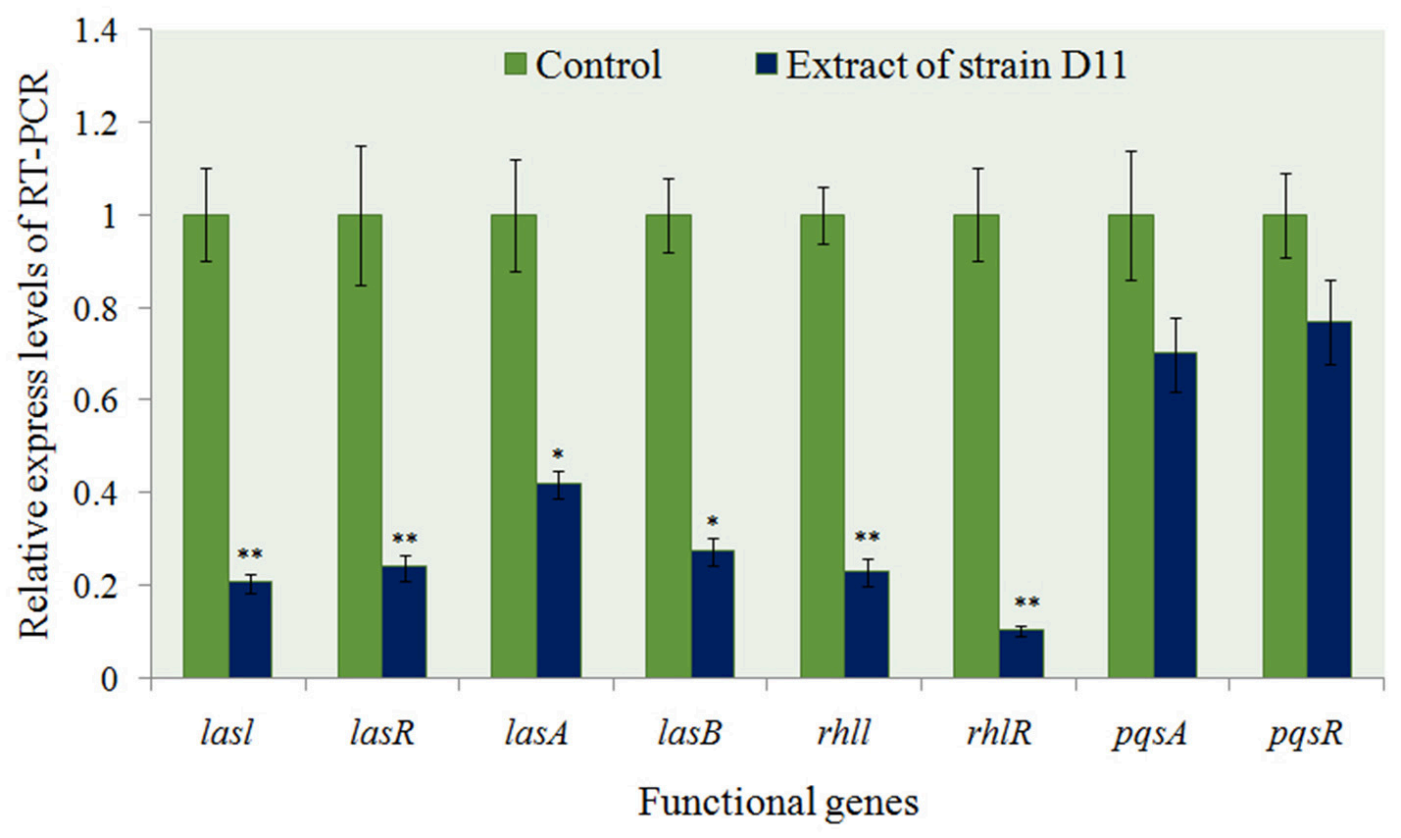
Expression profiling of some anti-QS regulatory genes from *P. aeruginosa* PAO1 with D11 extract measured by real-time PCR. The mRNA expression of these genes in the absence of extract served as a control. Results are based on three independent experiments and error bars represent means ± SD (*n* = 3). Asterisks indicate a statistically significant difference (^*^P < 0.05; ^**^P < 0.01) between experimental groups and control groups.

The transcriptional level of eight specific genes (*lasI, lasR, lasA, lasB, rhlI, rhlR, pqsA*, and *pqsR*) encoding putative biofilm-forming and QS factors was determined by RT-PCR in 24 h-old *P. aeruginosa* PAO1 cultures with extract and *P. aeruginosa* PAO1 cultures with methanol only as control. Approximately 2.5- to 5.1-fold down-regulation of the genes *lasI, lasR, lasA*, and *lasB* [responsible for acyl-homoserine lactone (AHL)-based biofilm formation] were observed in *P. aeruginosa* PAO1 cultured with the extract (*P* < 0.05 or *P* < 0.01) (Figure 8). Two virulence-related genes (*rhlI* and *rhlR*) also showed a significant decrease (72.3 and 88.5%, respectively) in expression level (*P* < 0.01). These results indicated that the general trend in expression for specific genes was similar between RT-PCR and biofilm state. As for the *Pseudomonas* quinolone signal (PQS) system in *P. aeruginosa* PAO1, there were no obvious differences between the experimental groups and the control groups (Figure 8).

### Bio-information from the whole genome of strain D11

The whole genome of strain D11 comprised 5,392,014 nucleotides and the G+C content was 44.69%. It contains 71 contigs with an N50 contig length of 126,438 bp. The whole genome encodes 76 tRNA and 17 rRNA genes. The genome predicted a total of 4522 genes with 3738 protein-coding genes (Supplementary Table 1). Based on functional categories of COG (http://www.ncbi.nlm.nih.gov/COG/), a total of 593 genes were annotated to be participating in carbohydrate and amino acid metabolism, another 1018 genes were predicted to have general functions (Supplementary Figures 1, 2). In addition, 285 genes were predicted to encode signal transduction molecules.

In addition, we analyzed the candidate genes related to homocysteine thiolactone production. Homocysteine, an intermediate compound in the methionine metabolic cycle, is an amino acid that includes a thiol group. The homocysteine thiolactone forms adducts through irreversible reactions with epsilon-NH_2_ groups of lysine residues. We found several methionine-related genes (*metI, metC, metF, metE*, and *mdh*) (Supplementary Figure 3) located in the upstream position (contig 1), these genes showed relatively high sequence identity to another species of the same genus, *Staphylococcus aureus* (GenBank accession numbers SACOL0431-ACOL0427) (Schoenfelder et al., 2013). Our analysis predicts the presence of these genes identified in isolate D11 might be involved in methionine (or its intermediate product homocysteine thiolactone) biosynthesis. However, the corresponding mutants need to be constructed in the future in order to confirm this assumption.

## Discussion

Among the marine environment, microorganisms and their metabolic products are a crucial source for the discovery of novel anti-QS compounds (Dong and Zhang, 2005; Choo et al., 2006; Dobretsov et al., 2006). Most of studies published on the production of QS inhibitors by marine bacteria have focused on bacteria that were collected from various niches, like surfaces, biofilms, and sediments (Teasdale et al., 2009, 2011). Indeed, many of the known anti-QS compounds have been discovered in sessile marine organisms such as sponges and microalgae that interact closely with bacteria (Stowe et al., 2011; Golberg et al., 2013). In the coral surface, Skindersoe et al. (2008) demonstrated that a large number of symbiotic microbes along the Great Barrier Reef corals possess anti-QS abilities. In addition, many studies were recently published indicating that QS inhibitors may be a frequently occurring feature in coral culturable bacteria such as *Bacillus* sp. and *Vibrio* sp. (Kanagasabhapathy et al., 2009; Thenmozhi et al., 2009; Nithya and Pandian, 2010a; Romero et al., 2012). These examples indicate that coral-derived bacteria may be potential sources of anti-QS compounds. In this work, approximately 15% of isolates from the hard coral species exhibited anti-QS activity. Among them, five strains (including *S. hominis* D11) were found to have significant anti-QS activity, supporting the hypothesis of Certner and Vollmer (2018), i.e., coral microbiota is a vast natural reservoir for developing new anti-QS substances.

Among the screened anti-QS bacteria, *S. hominis* D11 presented the most apparent opaque halo (growth of reporter strain with pigment inhibition) surrounding the isolate (Figure 1). The correlating results of violacein production (Figure 2B) further proved that the screened strains possess anti-QS activity against *C. violaceum* ATCC 12472. This inhibition activity seems similar to that of halogenated furanone, which inhibit QS function by interfering with *luxS*- or AI-2 system (Ren et al., 2002; Huang, 2009). Previously, some studies demonstrated that anti-QS activity and antimicrobial activity may co-occur (Busetti et al., 2014; Abudoleh and Mahasneh, 2017). In order to rule out the possibility that the inhibitory effect on the production of purple pigment was due to an antimicrobial effect, growth experiments with different test strains were performed, and no significant difference was observed among the experimental and control groups (Figure 2A). These results indicated that absence of violacein is mainly caused by QS disruption.

Accumulating data evidence that AHL-dependent QS is a key factor for formation of biofilms, indicating that anti-QS substances can inhibit biofilm development. Research by Adonizio et al. (2008) and Nithya et al. (2010b) suggest that *P. aeruginosa* PAO1 biofilm maturation can be inhibited by marine-derived bacterial species *Callistemon viminalis* and *Bacillus pumilus* S8-07, respectively. Teasdale et al. (2009) also found that the anti-QS properties exhibited by the marine bacterium *Halobacillus salinus* C42 were present in the solvent phase, in which the solvent was ethyl acetate. In this study, we found that addition of D11 organic extract resulted in significant reduction in the *P. aeruginosa* PAO1 biofilm (Figure 3A), indicating that active anti-QS extract may contain non-enzymatic compounds. In addition, inhibition of the AHL-dependent QS system by bacterial extracts was also observed. We speculated that the activity substance was associated with AHL analogs that acted as AHL-antagonists by competing with AHL for receptor binding and eventually inhibit biofilm formation of *P. aeruginosa* PAO1. In order to test the hypothesis and elucidate the possible mechanisms responsible for the inhibitory properties, related studies aimed at purifying and characterizing D11 extracts were carried out. After HPLC-MS-NMR analysis, we determined from the chemical structure that the activity substance was DL-homocysteine thiolactone (Figure 6D). Interestingly, this compound is very similar to homoserine lactone (HSL), produced from hydrolysis of AHLs, a common QS signal molecule (McInnis and Blackwell, 2011). This result supported our hypothesis along with further confirmation that AHL-based analogs have been extensively developed as QS modulators or anti-biofilm agents (Melvin et al., 2016). Interestingly, the violacein inhibition and anti-QS activities were confirmed by using the pure commercial DL-homocysteine thiolactone substance, which leads to the proposal that the QS inhibitory activity produced by strain D11 is homocysteine thiolactone. This is the first report of anti-biofilm activity of DL-homocysteine thiolactone on *P. aeruginosa*; further study is required to develop this substance as an anti-bacterial agent for treatment of the biofilm-forming pathogenic bacteria.

Quorum-sensing genes are key regulators of biofilm development, various extracellular virulence factors, luminescence and the antibiotic resistance of bacterial pathogens (Schuster and Greenberg, 2006; deKievit, 2009; Sharma et al., 2014). There are three well-characterized QS networks that have been identified in *P. aeruginosa*: *las*-, *rhl*-, and *pqs*-pathways. The three pathways utilize the corresponding AHLs: respectively, N-3-oxo-dodecanoyl homoserine-lactone (3OC12-HSL), N-butanoylhomoserine lactone (C4-HSL), and 2-heptyl-3-hydroxyl-4-quinolone (*Pseudomonas* quinolone signal, or PQS) (Zhang and Dong, 2004). In these systems, *lasI* and *rhlI* are involved in autoinducer synthesis, and *lasR* and *rhlR* code for transcriptional activators (Sharma et al., 2014). In our work, significantly reduced *lasI, lasR, rhlI*, and *rhlR* expression were observed (Figure 8), indicating that the D11 extract (DL-homocysteine thiolactone) has the ability to inhibit *lasR* and *rhlR* regulatory systems. It perhaps suggests that the mechanism for QS inhibition is via interaction with both *las* and *rhl* receptors. This result was also found by Vattem et al. (2007) who achieved the same result with extracts of *Kigelia africana*. In addition, unlike the chemically synthesized QS inhibitors, such as furanone, cyclopentanols and furanone derivatives (Givskov et al., 1996; Hentzer et al., 2002; Ishida et al., 2007; Geske et al., 2008; Kim et al., 2008), the natural compounds (for instance, DL-homocysteine thiolactone) have several advantages, including low toxicity and being environment-friendly. These features expand their potential utility in the biomedical field as natural QS inhibitors. In addition to anti-biofilm activity, strain D11 also inhibited the production of *P. aeruginosa* PAO1 virulence factors such as *las* genes (Figure 8). These results are similar to findings by Park et al. (2005), which showed that the *Streptomyces* strain M664 produces an AHL-degrading acylase enzyme that degrades AHL-regulated elastase and total and *lasA* proteases by 43–50%. Musthafa et al. (2011) also demonstrated that the marine-derived *Bacillus* sp. SS4 inhibited AHL-regulated production of *P. aeruginosa* virulence factors. In addition, our previous work found that the inhibition of elastase activity and siderophore production by *Rhizobium* sp. NAO1 occurs via interference with QS activity because these virulence factors are under the control of the *las-*coding gene systems (Chang et al., 2017).

For the whole-genome data of strain D11 (*S. hominis*), genome annotation on predicted genes was carried out by BLAST searches against anon-redundant protein sequence database and other databases available online, such as COG and KEGG. Based on the functional categories and gene annotation analysis (Supplementary Figure 2), 216 genes of strain D11 are involved in carbohydrate metabolism and 472 genes participate in nitrogen utilization and energy conversion, which allows this microorganism to adapt to coral-bacteria symbiosis. After gene annotation analysis, 377 genes were related to amino acids processing. Potentially, these genes are a key feature of strain D11 that enable it to biosynthesize all kinds of amino acids, including an intermediate compound (homocysteine) in methionine metabolism. For methionine or related by-products (such as homocysteine thiolactone), several *metI/E/F*-encoding genes were predicted to be located at contig1 (Supplementary Figure 3). These genes showed relatively high identity to another species of the same genus, *Staphylococcus aureus* (Grundy and Henkin, 1998). Our results further support the previous viewpoint, i.e., many microorganisms are able to synthesize methionine *de novo* and staphylococci employ the trans-sulfuration pathway to generate methionine (Rodionov et al., 2004). In this work, the whole-genome sequence of strain D11 provides deeper understanding of the molecular mechanism of the anti-QS ability of strain D11, and also may facilitate insights into the active product biosynthesis process.

## Conclusions

In this work, we uncovered the anti-QS activity of a marine bacterial species isolated from the coral *Pocillopora damicornis*. The extract of strain D11 (*S. hominis*) was antagonistic to *P. aeruginosa* PAO1 QS and affected QS-regulated functional genes, including those involved in biofilm formation and virulence production. It is possible that the analog molecule DL-homocysteine thiolactone produced by strain D11 (*S. hominis*) competed with the auto-inducers produced by *P. aeruginosa* PAO1. Interestingly, DL-homocysteine thiolactone did not affect the growth of *P. aeruginosa* PAO1. These characteristics may accelerate development of QS inhibitors with broad-spectrum activity, and facilitate the discovery of novel drugs with greater efficacy to deal with bacterial infections in the current post-antibiotic era.

## Author contributions

Z-PM and JZ performed the experiments and drafting of the manuscript. YS and Z-HC acquired and analyzed data. Z-JL prepared figures and tables. YW and G-HL completed critical revision.

### Conflict of interest statement

The authors declare that the research was conducted in the absence of any commercial or financial relationships that could be construed as a potential conflict of interest.
