# Supplementary material for: Anti-quorum Sensing Activities of Selected Coral Symbiotic Bacterial Extracts From the South China Sea
Source: Front Cell Infect Microbiol. 2018 May 8;8:144. doi: 10.3389/fcimb.2018.00144 (PMC5951975; doi:10.3389/fcimb.2018.00144)
Supplement: Supplementary file 1 [file Presentation_1.PDF]

Supplementary Materials

Figure S1. Circular map for the whole genome of *S. hominis* strain D11. From the outside to the center: encoding genes, predicted CDSs transcribed in the clockwise (or counterclockwise) direction, ncRNA, GC percent (%), and GC skew (G + C/G-C) in a 1000-bp window.

Figure S2. Function category distribution of *S. hominis* D11 (based on COG function statistics).

Figure S3. Determination of a leader RNA transcript upstream of methionine or intermediate compound (homocysteine) biosynthesis genes. Overview of the genomic organization of the methionine biosynthesis operon. A transcript (blue arrow) was detected upstream of *metI*.

Table S1. Genome features of *Staphylococcus hominis* D11.

Attributes	Values
Genome size	5.39 M
GC content %	44.69%
Number of Contigs	71
Total contig size	4,878,573 bp
Largest contig	275,433 bp
Scafflods	6
Protein coning genes	4522
tRNAs	76
rRNAs	17
Minisatellite DNA	37
Microsatellite DNA	0
Genes with a predicted function	21
Potential methionine encoding site	Contig 1
Encoding-methionine biosynthesis gene length	469 bp
