# Supplementary figures and images for: Anti-quorum Sensing Activities of Selected Coral Symbiotic Bacterial Extracts From the South China Sea
Source: Front Cell Infect Microbiol. 2018 May 8;8:144. doi: 10.3389/fcimb.2018.00144 (PMC5951975; doi:10.3389/fcimb.2018.00144)

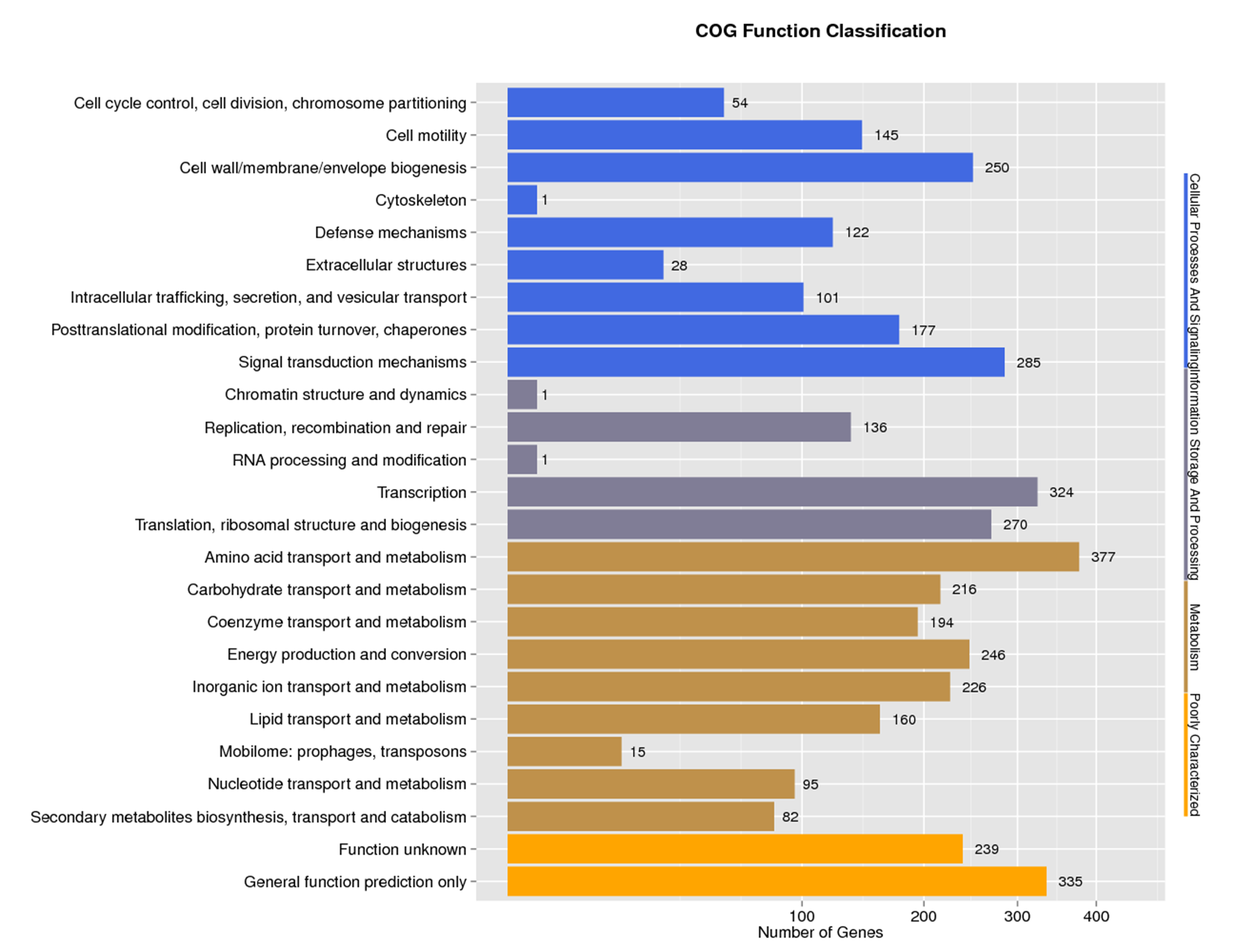

Supplement: Supplementary file 3 [file Image_2.TIF]

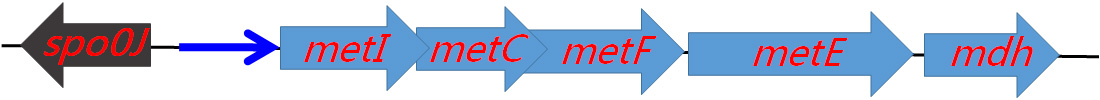

Supplement: Supplementary file 4 [file Image_3.JPEG]
